# Transfection of Vein Grafts with Early Growth Response Factor-1 Oligodeoxynucleotide Decoy: Effects on Stem-Cell Genes and Toll-like Receptor-Mediated Inflammation

**DOI:** 10.3390/ijms242115866

**Published:** 2023-11-01

**Authors:** Konstantinos S. Mylonas, Michail Peroulis, Alkistis Kapelouzou

**Affiliations:** 1 Department of Cardiac Surgery, Onassis Cardiac Surgery Center, 17674 Athens, Greece; 2 Department of Surgery, Vascular Surgery Unit, Faculty of Medicine, University of Ioannina, 45110 Ioannina, Greece; 3 Clinical, Experimental Surgery & Translational Research, Biomedical Research Foundation Academy of Athens, 11527 Athens, Greece; akapel@bioacademy.gr

**Keywords:** Egr-1, decoy, gene therapy, vein graft, intimal hyperplasia, TLR, MYD88, stem cell genes, KLF4, NANOG, HOXA5, HIF1α

## Abstract

The long-term patency of vein grafts is challenged by intimal hyperplasia. We sought to explore the intricate relationships between the transcription factor Egr-1, toll-like receptors (TLRs), and stem cell genes and also assessed oligodeoxynucleotide decoys (ODNs) as a strategy to prevent vein graft failures. A total of 42 New Zealand white rabbits were fed hyperlipidemic chow and classified into three groups. A double-stranded Egr-1 ODN was synthesized and infused in vein grafts prior to anastomosis with the common carotid artery. All vein grafts were retrieved at the conclusion of the predefined experimental period. Real-time quantitative polymerase chain reaction was performed to estimate expression patterns for several genes of interest. MYD88, TLR2-4, TLR8, NF-kB, TNF-α, IFNβ, and IFNγ; chemokines CCL4, CCL20, CCR2; numerous interleukins; and stem cell genes KFL4, NANOG, HOXA5, and HIF1α were universally downregulated in the ODN arm compared with the controls. By understanding these multifaceted interactions, our study offers actionable insights that may pave the way for innovative interventions in vascular reconstructions.

## 1. Introduction

Vascular reconstruction has heavily relied on vein grafts following the seminal work of Carrel and Guthrie in 1906 [1]. Despite technological advancements in synthetic bypass conduits, vein grafts remain paramount in peripheral vascular surgery. Similarly, venous conduits continue to be routinely utilized in multi-vessel coronary artery bypass graft surgery (CABG) [2]. The primary challenge to their long-term efficacy is the development of intimal hyperplasia, a vexing problem that has resisted resolution by various interventions, including pharmacologic agents, external sheaths, stents, and even various forms of radiation [3,4].

Central to this issue is Egr-1 (early growth response factor-1), a zinc finger transcription factor expressed in the vascular wall’s diverse cellular makeup [5]. Its activation triggers a cascade of proinflammatory gene expressions, including nuclear factor kappa B (NF-kB) and tumor necrosis factor α (TNFα), as well as various interferons (IFNs), interleukins (ILs), and endothelial adhesion molecules [6,7]. This cascade is extensively involved in the proliferative response leading to intimal hyperplasia. In our previous research, we found that the utilization of Egr-1 decoy oligodeoxynucleotide (ODN) exerts a marked effect on vein grafts, notably inhibiting Egr-1 mRNA concentration and restraining cellular proliferation [8]. This significant reduction in intimal hyperplasia became evident within the initial six-week postoperative period and subsequently demonstrated a persistent effect.

In the context of toll-like receptor (TLR) signaling pathways, a two-fold categorization emerges: MYD88-dependent, culminating in NF-κB activation, and TRIF-dependent. These pathways synergize in inducing proinflammatory cytokines, suggesting a potential connection to intimal hyperplasia. TLR activation, particularly through the MYD88 pathway, may provide another piece of the puzzle in understanding the mechanistic underpinnings of vein graft failure.

Emerging evidence has also revealed that the expression of various stem cell genes (including KLF4 (Krüppel-like factor 4), HOXA5, NANOG, and HIF1α (hypoxia-inducible factor 1-alpha)) can be dysregulated by shear stress [9,10,11]. These genetic alterations may potentially orchestrate specific patterns in venous graft failure, including the development of neointimal hyperplasia [12,13,14].

The present study navigates the intricate terrain of Egr-1, ODN, TLRs, and stem cell genes, formulating a coherent analysis of their interrelations. By probing these multifaceted interactions, we hope to shed light on innovative strategies to manage vein graft failures.

## 2. Results

No morbidity or fatalities were noted in any of the study subjects. Supplemental Table S1 provides a detailed outline of mRNA expression metrics for all target genes.

### 2.1. TLR and MYD88

**Figure 1:**
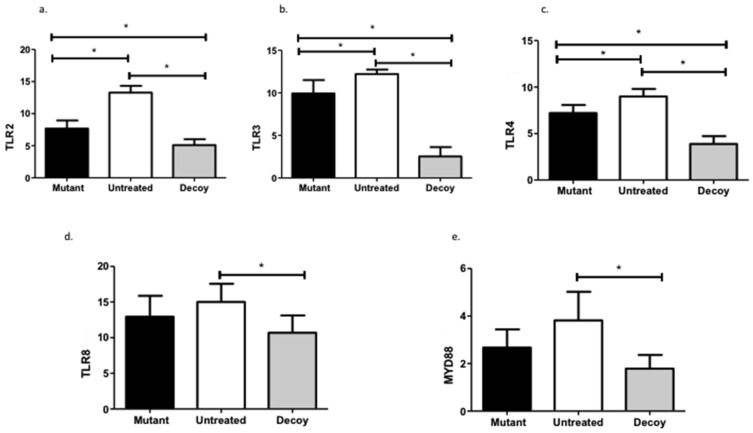
TLR and MYD88 expression patterns for NF-kB, TNF-a, and interferons. MYD88: myeloid differentiation primary response 88; TLR: toll-like receptor; *: statistically significant comparisons.

TLR2 was significantly downregulated in the ODN arm compared with both the control (MD: 8.17, 95% CI: 6.54–9.80) and the mutated decoy (MD: 2.56, 95% CI: 0.93–4.20) subgroups. TLR3 was also significantly reduced in ODN rabbits versus their untreated (MD: 9.68, 95% CI: 7.95–11.42) and mutated (MD: 7.40, 95% CI: 5.67–9.13) counterparts. Similarly, TLR4 expression curtailed following ODN treatment compared with both controls (MD: 5.11, 95% CI: 3.85–6.38) and mutated analogs (MD: 3.33, 95% CI: 2.06–4.59). On the other hand, TLR8 (MD: 4.31, 95% CI: 0.34–8.28) was only downregulated in the decoy group compared with the untreated controls. MYD88 followed the same expression pattern (MD: 2.03, 95 CI: 0.70–3.36) (Figure 1 TLR and MYD88 expression patterns: MYD88: myeloid differentiation primary response 88; TLR: toll-like receptor; *: statistically significant comparisons).

### 2.2. NF-kB, TNF-a, and Interferons

**Figure 2:**
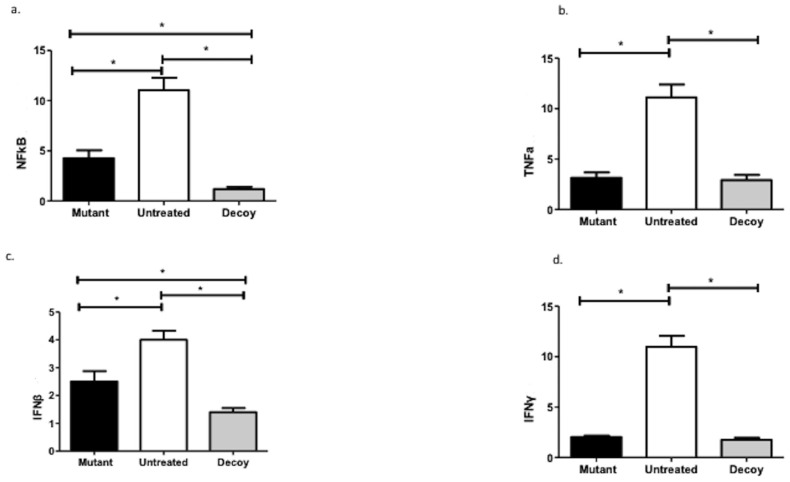
NF-kB, TNF-a, and interferon expression patterns. NF-kB: nuclear factor kappa-light-chain-enhancer of activated B cells; TNF-a: tumor necrosis factor-alpha; IFN: interferon; *: statistically significant comparisons.

Decoy infusion led to statistically significant reductions in NF-kB (MD: 28.01, 95% CI: 8.55–11.13; MD: 3.07, 95% CI: 1.78–4.36) and IFNβ (MD: 2.59, 95% CI: 2.15–3.04; MD: 1.10, 95%: 0.66–1.55) levels compared with both the untreated and the mutated arms. On the other hand, TNF-α (MD: 8.19, 95%: 6.92–9.46) and IFNγ (MD: 9.35, 95% CI: 8.23–10.48) were only attenuated in the decoy group compared with the controls (Figure 2).

### 2.3. Chemokines

**Figure 3:**
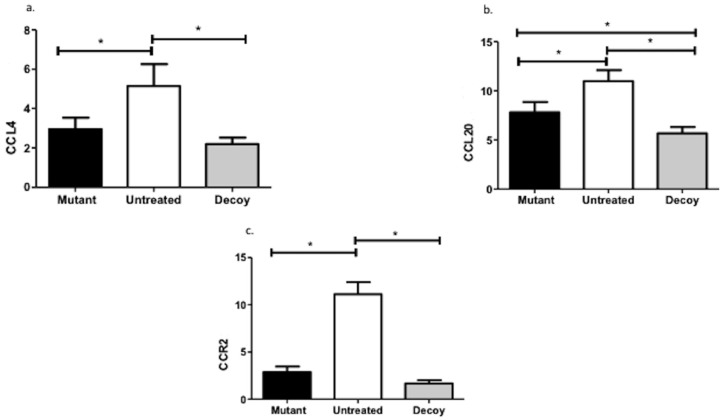
Chemokine expression patterns. CCL4: C-C chemokine ligand; CCL20: chemokine (C-C motif) ligand 20; CCR2: C-C chemokine receptor type 2; *: statistically significant comparisons.

CCL20 expression was substantially decreased with ODN inoculation compared with not only the controls (MD: 13.60, 95% CI: 3.88–6.75) but also the mutated decoys (MD: 2.14, 95% CI: 0.70–3.57). Conversely, ODN treatment decreased CCL4 (MD: 2.94, 95% CI: 1.81–4.06) and CCR2 (MD: 9.43, 95% CI: 8.18–10.68) levels only in comparison with untreated animals (Figure 3).

### 2.4. Interleukins

**Figure 4:**
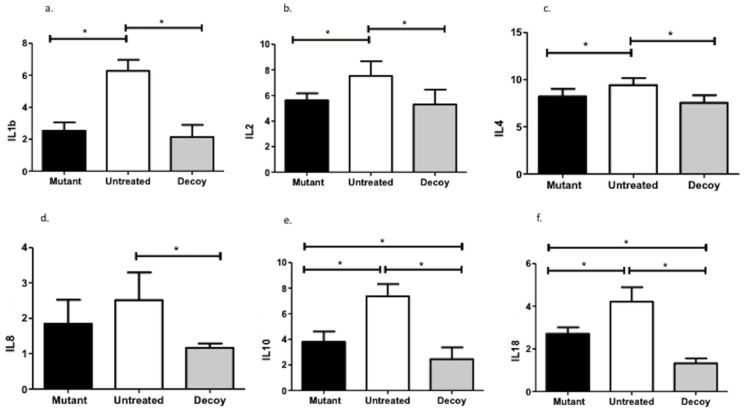
Interleukin expression patterns. IL: interleukin; *: statistically significant comparisons.

IL10 (MD: 4.91, 95% CI: 3.56–6.25; MD: 1.34, 95% CI: 0.10–2.69) and IL18 production (MD: 2.88, 95% CI: 2.21–3.56; MD:1.38, 95% CI: 0.71–2.064) was more potently reduced with ODN compared with both controls and mutated inoculations. That said, decoy infusion attenuated IL1b (MD: 4.12, 95% CI: 3.13–5.12), IL2 (MD: 2.22, 95% CI: 0.73–3.71), IL4 (MD: 1.87, 95% CI: 0.69–3.05), and IL8 (MD: 1.35, 95% CI: 0.44–2.25) only compared with untreated subjects (Figure 4).

### 2.5. Stem Cell Genes

**Figure 5:**
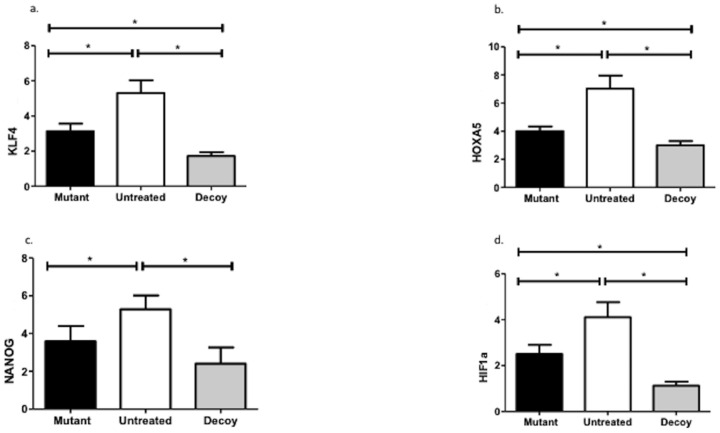
Stem cell gene expression patterns. KLF4: Krüppel-like factor 4; HIF1α: hypoxia-inducible factor 1-alpha; *: statistically significant comparisons.

Decoy inoculation significantly downregulated KLF4 (MD: 3.59, 95% CI: 2.83–4.34; MD; 1.41, 95% CI: 0.65–2.16), HOXA5 (MD: 4.02, 95% CI: 3.11–4.92; MD: 1.07, 95% CI: 1.91), and HIF1α (MD: 2.98, 95% CI: 2.30–3.66; MD: 1.38, 95% CI: 0.70–2.06) compared with both the untreated and mutated cohorts. However, NANOG expression (MD: 2.88, 95% CI: 1.67–4.08) was reduced in the decoy group only compared with untreated controls (Figure 5).

### 2.6. Correlations

To better elucidate important pattern interplays, we performed an analysis of correlation coefficients. 

#### 2.6.1. ODN-Infused Vein Grafts

A statistically significant positive association was observed between TLR3 and CCL4 (r = 0.81, *p* = 0.04), as well as IL4 (r = 0.91, *p* = 0.01). TLR4 expression was positively associated with CCL4 (r = 0.84, *p* = 0.03) but negatively with CCL20 (r = −0.95, *p* < 0.01) and IL10 (r = −0.84, *p* = 0.03). CCL4 expression was inversely correlated with IL18 (r = −0.85, *p* = 0.03). Lastly, IFNγ levels were negatively associated with HOXA5 (r = −0.82, *p* = 0.04) but positively with NANOG (r = 0.87, *p* = 0.02) (Supplemental Figure S2).

#### 2.6.2. Other Correlations

Supplemental Figures S3 and S4 provide a comprehensive overview of all statistically significant correlations that were noted in the control and mutated subgroups.

## 3. Discussion

Intimal hyperplasia in venous grafts is a complex process that is influenced by versatile proinflammatory stimuli. It involves intricate intracellular pathways with both inhibitory and pro-proliferative proteins. Therefore, targeting a transcription factor that impacts multiple pathways could provide a strategic leverage point for graft failure prevention and treatment [15]. Transcription factors such as E2F, nuclear factor kappa B (NF-kB), activator protein-1 (AP-1), and the CCAAT/enhancer binding protein (C/EBP) have been identified as potential targets for decoy ODNs [16,17,18,19]. 

Egr-1 is prevalent in a range of cell types within the vascular wall, notably endothelial cells, vascular smooth muscle cells, monocytes, and macrophages. Its expression can be triggered by various factors associated with vascular disease progression, such as shear and mechanical stresses, hypoxic conditions, insulin, glucose, angiotensin II, and immediate arterial injuries [5,6,7]. Research has shown that the intra-operative transfection of E2F decoy ODNs into jugular vein-to-carotid artery interposition grafts successfully can inhibit neointimal hyperplasia and atherosclerotic plaque formation over a 6-month period in hypercholesterolemic rabbits. This also led to reduced occlusions, revisions, and arterial stenoses in human saphenous vein grafts at 12 months post-coronary surgery [20]. Nevertheless, E2F decoy treatment (edifoligide) has failed to demonstrate efficacy beyond that of a placebo in preventing graft failure or major adverse cardiac events 12 to 18 months after CABG [21].

Our previous work benchmarked the efficacy of Egr-1 ODN in the treatment of vein grafts [8]. DAPI staining confirmed the successful introduction of the ODN into the nuclei of venous endothelial and smooth muscle cells. Specifically, Egr-1 decoy ODN transfection resulted in the substantial inhibition of Egr-1 mRNA concentration and cellular proliferation, with reductions ranging from 34% to 60%. Moreover, it significantly reduced intimal hyperplasia, with thickness reductions of up to 50%. These effects were apparent within the first 6 weeks of decoy treatment and persisted thereafter [14]. 

In the present analysis, we built upon that preliminary experience by exploring expression pattern modifications of key cardiovascular stem cell genes, TLRs/ MYD88, and numerous other proinflammatory cytokines. Of note, decoy inoculation dramatically reduced the expression of all examined mediators, including TNFa; NF-kB; and chemokines CCL4, CCL20, and CCR2. The production of numerous interleukins (IL1b, IL2, IL4, IL8, IL10, IL18) and interferons (IFNβ, IFNγ) was also uniformly curtailed with ODN infusions. The pronounced effect of the present decoy strategy reinforces the potential of transcription factors as valuable targets for addressing cardiovascular inflammation.

Furthermore, ODN treatment effectively reduced the expression of TLR2, TLR3, TLR4, TLR8, and MYD88. Interestingly, the overexpression of TLR3 and TLR4 has been implicated in the severity of coronary artery disease [22]. A growing body of literature has also shown that the downregulation of MYD88 or TLR2/4 leads to impaired macrophage function, prevents the mesenchymal transition of smooth muscle cells, and diminishes CCL2/CCL4 levels [23]. The release of reactive oxygen species (ROS) is also hindered in the setting of MYD88 downregulation [24,25]. Additionally, MYD88 attenuation appears to reduce the secretion of interleukins 1b/6 and other chemokines, as well as the expression of adhesion molecules (i.e., ICAM1 and VCAM1). Conversely, the activation of MYD88-driven pathways in endothelial cells in response to injury induces the expression of the atherogenic proprotein convertase subtilisin/kexin type 9 (PCSK9) [26].

Last but not least, decoy infusion extensively downregulated KLF4, NANOG, HOXA5, and HIF1α. As previously conveyed by our group, a hyperlipidemic diet induces the expression of the KLF4 gene, which, in turn, dysregulates nitric oxide synthesis, VE-cadherin transcription, intercellular connections, and macrophage/monocyte differentiation [9,27,28,29,30,31]. Similarly, hindering the overexpression of NANOG, HOXA5, and HIF1α diminishes the proliferative, migratory, and anti-apoptotic capabilities of vascular SMCs [32,33,34,35]. HIF1α downregulation also curtails the production of adhesion molecules such as CXCL1, ICAM-1, and VCAM-1 [36,37]. Similarly, NANOG diminishment prevents vascular calcification and preserves VE-cadherin in adherens junctions [38].

It is worth noting that both the original and mutated ODNs are designed to interact with Egr-1. While the mutated form does have sequence alterations, its foundational design is derived from the original ODN. As such, it is not surprising for them to exhibit overlapping activities and influence the expression of some of the same genes. However, inherent differences between them also lead to unique interactions with certain pathways. Indeed, the majority of the target genes were downregulated only after Egr-1 ODN treatment. These include TLR2, TLR3, TLR4, NF-kB, IFNβ, chemokine CCL20, Il10, and most stem cell genes (KLF4, HOXA5, HIF1α). Although large-scale randomized clinical trials are needed to validate these data in human patients, our findings are promising. Indeed, the present approach is straightforward, cost-effective, and does not necessitate specialized equipment. The technique theoretically tackles the stimuli of early failure due to harvest trauma (i.e., endothelial injury and graft ischemia related to vasa vasorum disruption). That said, certain limitations should be discussed. First, our research centered on examining mRNA expression patterns. While the evaluation of Egr-1’s binding activity following Egr-1 decoy ODN treatment was not within our IRB-approved study’s remit, it is a direction we plan to explore in upcoming studies.

Second, our investigation primarily centers on whole tissue, even though we recognize that Egr-1 is not limited to the endothelium but is also expressed in SMCs and macrophage-type cells. Moving forward, we will attempt to delve into the distinct roles Egr-1 might play in each individual cell type. Furthermore, graft transfection with short-acting decoys cannot address patient-related risk factors, including tobacco smoking, dyslipidemia, or poorly controlled diabetes mellitus. Indisputably, following the gold-standard medical treatment is key to improving graft longevity. In the future, improving transfection techniques, employing decoys with prolonged action (such as short interfering RNA), and devising chimeric ODNs that target more than one transcription factor could potentially revolutionize this field. 

## 4. Materials and Methods

### 4.1. Test Subjects and Oligonucleotides

Our experimental model was previously described and validated [8]. In brief, a total of 42 male New Zealand white rabbits (Oryctolagus cuniculus) within the age range of 12 to 15 weeks and weighing between 3 and 3.5 kg were engaged in the present study. Participating rabbits were placed on a diet that consisted of 2% cholesterol, commencing one week prior to surgical procedures and lasting until vein graft collection. 

A phosphorothioate-stabilized, double-stranded Egr-1 oligodeoxynucleotide decoy (ODN) was synthesized, containing two copies of the sequence 5′-GCGGGGGCG-3′, representing the Egr-1 consensus binding sequences (Invitrogen Corporation, Carlsbad, CA, USA). A mutant Egr-1 decoy ODN was used as a negative control and contained the sequence 5′-GCTAGGGCG-3′. Both ODNs were dissolved at a concentration of 40 mmol/L in normal saline solution. 

### 4.2. Operative Protocol

Preoperative care included intramuscular administration of xylazine and ketamine, followed by tracheal intubation and mechanical ventilation. Isoflurane was utilized to sustain anesthesia, and the auricular vein was used for administering fluids. 

A 2.5 cm segment of the right external jugular vein was harvested through a midline neck incision using a “no-touch” approach. The vein graft underwent cannulation and was then flushed with saline, secured, and encased in a plastic sheath. Infusion and pressurization of ODN solution (decoy, mutant decoy, or control) were performed at 300 mmHg for 20 min with the use of a balloon inflation device (Styker Diskmonitor, Kalamazoo, MI, USA).

Heparin (200 IU/kg) was administered intravenously, and the ipsilateral common carotid artery was clamped proximally and distally and divided. The vein graft was anastomosed to the divided artery in a reversed end-to-end fashion with interrupted 7/0 polypropylene sutures. Mean diameter measurements for common carotid arteries and venous grafts were 2.03 ± 0.29 mm and 1.78 ± 0.21 mm, respectively. No mismatch was noted at the completion of the anastomosis. After wound closure, all subjects were transferred to the Intensive Care Unit with ongoing monitoring.

### 4.3. Study Groups

For the purposes of the experiment, the rabbits were classified into three distinct groups (Supplemental Figure S1). Group A, comprising 18 animals, was treated with the mutant decoy ODN (40 mmol^−1^). Group B, consisting of 18 animals, had no treatment. Group C, including six animals, was treated with a fluorescein isothiocyanate (FITC)-labeled decoy.

The timing of sacrifice was set differently between the groups, with animals in Groups A and B being divided into thirds for sacrifice at 48 h, 6 weeks, and 12 weeks. Post-surgical examination included the dissection, fixing, and processing of grafts excised after 6 and 12 weeks, followed by paraffin embedding. Grafts removed after 48 h were used in quantitative real-time reverse transcription polymerase chain reaction (RT-PCR) to gauge the expression of several genes of interest.

After a period of 48 h, the animals in Group C were sacrificed, with their patency being confirmed through the utilization of a color Duplex scan. The vein grafts from these subjects underwent homogenization and examination using RT-PCR to ascertain the expression level of key stem cell genes, TLRs, MYD88, and proinflammatory cytokines.

### 4.4. mRNA Analysis

RT-PCR was performed to estimate expression patterns for several genes of interest in extracted vein grafts [12]. The purity of the extracted RNA was measured with a spectrophotometer. RNA was reverse-transcribed, and the resulting complementary DNA (cDNA) was synthesized using the M-MLV Reverse transcriptase (Thermo Fisher Scientific, Boston, MA, USA). Real-time quantitative polymerase chain reaction was performed using SYBR Green (Invitrogen, Life Technologies, Carlsbad, CA, USA), according to our institutional protocol [13]. 

All primers for β-actin, TLR2, TLR3, TLR4, TLR8, MYD88, NF-kB, CCL4, CCL20, CCR2, IFN-β, IFN-γ, TNF-α, IL-1b, IL-2, IL-4, IL-8, IL10, IL-18, KLF4, HOXA5, NANOG, and HIF1α were synthesized with TIB Molbiol (Syntheselabor GmbH, Berlin, Germany). The sequences of primer pairs were designed with the Beacon Designer V7.0 software (Premier Biosoft International, Palo Alto, CA, USA). Primer sequences are provided in Supplemental Table S2. Each 20 μL qPCR reaction consisted of 2 μL of cDNA (transcribed from 20 ng of total RNA), primers at 200 nM, and 10 μL of SYBR Green, following institutional protocols. QRT-PCR was performed using a thermocycler (LightCycler 480; Roche, Mannheim, Germany) under the following specific cycling conditions: 40 cycles of 95 °C × 30 s, 60 °C × 40 s, 72 °C × 40 s, following an introductory denaturation at 95 °C that lasted for ten minutes. Both the RT and qPCR reactions were repeated three times. The amplification signals from each target gene were normalized to β-actin (housekeeping gene). The 2^−ΔΔCq^ method was used for quantification of the target gene [14].

### 4.5. Statistical Analysis

All values were expressed as mean ± SD (standard deviation). Mean differences (MDs) with 95% confidence intervals (95% CI) were calculated. Statistical analysis was performed using one-way analysis of variance (ANOVA) followed by Tukey’s Multiple Comparison Test. Correlation between measured parameters was assessed with Pearson analysis. *p*-values were two-sided, and statistical significance was set at *p* < 0.05. All statistical calculations were performed using GraphPad Prism version 4.03 (Graphpad Inc., La Jolla, CA, USA).

## 5. Conclusions

In the present study, the transfection of vein grafts with Egr-1 ODN diminished the production of proinflammatory mediators and profoundly downregulated the expression of MYD88, TLRs, and stem cell genes compared with the controls and mutated analogs. These insights provide novel targets for treatment and could be utilized to improve the longevity of venous grafts in cardiovascular surgery.

## Data Availability

Raw data may be provided by the authors upon request.

## Supplementary Materials

The supporting information can be downloaded at https://www.mdpi.com/article/10.3390/ijms242115866/s1.
