# Supplementary material for: Transfection of Vein Grafts with Early Growth Response Factor-1 Oligodeoxynucleotide Decoy: Effects on Stem-Cell Genes and Toll-like Receptor-Mediated Inflammation
Source: Int J Mol Sci. 2023 Nov 1;24(21):15866. doi: 10.3390/ijms242115866 (PMC10647335; doi:10.3390/ijms242115866)
Supplement: Supplementary file 1 [file ijms-24-15866-s001.zip › Supplemental Figure S3. Untreated correlations.pdf]

	MYD88	TLR2	TLR3	TLR4	TLR8	NFkB1	CCL4	CCL20	IFNγ	TNFa	ILb	IL2	IL4	IL8	IL10	IL18	KLF4	HOXA5	NANOG	H1F1a
MYD88																				
TLR2																				
TLR3																				
TLR4																				
TLR8																				
NFkB1																				
CCL4			0.016 (0.895)																	
CCL20																				
IFNγ								0.014 (0.902)												
TNFa																				
ILb																				
IL2																				
IL4					0.017 (0.891)					0.048 (0.815)										
IL8																				
IL10																				
IL18																				
KLF4								0.018 (0.860)	0.028 (0.861)											
HOXA5																				
NANOG																				
H1F1a												0.025 (0.868)		0.037 (0.839)						
