# Supplementary material for: Transfection of Vein Grafts with Early Growth Response Factor-1 Oligodeoxynucleotide Decoy: Effects on Stem-Cell Genes and Toll-like Receptor-Mediated Inflammation
Source: Int J Mol Sci. 2023 Nov 1;24(21):15866. doi: 10.3390/ijms242115866 (PMC10647335; doi:10.3390/ijms242115866)
Supplement: Supplementary file 1 [file ijms-24-15866-s001.zip › Supplemental Table S1. mRNA levels.pdf]

Table S1. Genes of interest: mRNA expression

Target genes		Mutant (group B)	Untreated (group D)	Decoy (group E)
**TLR2**	mean	7.672	13.28	5.103
	SD	1.267	1.062	0.909
**TLR3**	mean	9.933	12.21	2.527
	SD	1.576	0.533	1.105
**TLR4**	mean	7.208	8.993	3.877
	SD	0.874	0.814	0.838
**TLR8**	mean	12.93	15.01	10.70
	SD	2.936	2.552	2.419
**MYD88**	mean	2.675	3.820	1.787
	SD	0.762	1.203	0.576
**NFkB**	mean	4.273	11.04	1.198
	SD	0.783	1.249	0.218
**CCL4**	mean	2.958	5.142	2.198
	SD	0.581	1.115	0.323
**CCL20**	mean	7.818	11.00	5.677
	SD	1.052	1.113	0.639
**CCR2**	mean	2.88	11.11	1.680
	SD	0.583	1.275	0.335
**IFNb**	mean	2.508	4.000	1.403
	SD	0.369	0.327	0.142
**IFNγ**	mean	2.033	10.98	1.758
	SD	0.132	1.085	0.192
**TNFa**	mean	3.145	11.11	2.918
	SD	0.53	1.275	0.508
**IL1b**	mean	2.54	6.270	2.143
	SD	0.513	0.701	0.755
**IL2**	mean	562	7.520	5.295
	SD	0.548	1.148	1.163
**IL4**	mean	8.218	9.417	7.542
	SD	0.797	0.753	0.806
**IL8**	mean	1.847	2.515	1.165
	SD	0.679	0.783	0.122
**IL10**	mean	3.803	7.365	2.455
	SD	0.81	0.957	0.919
**IL18**	mean	2.713	4.212	1.325
	SD	0.298	0.68	0.235
**KLF4**	mean	3.135	5.312	1.722
	SD	0.429	0.726	0.224
**HOXA5**	mean	4.007	7.018	2.998
	SD	0.329	0.941	0.296
**NANOG**	mean	3.588	5.285	2.403
	SD	0.809	0.732	0.862
**H1F1α**	mean	2.510	4.105	1.125
	SD	0.394	0.657	0.171

*Myeloid differentiation primary response 88 (*MYD88*); Nuclear factor kappa-light-chain-enhancer of activated B cells (NF-κB); C-C chemokine ligand (CCL4); Chemokine (C-C motif) ligand 20 (CCL20); C-C chemokine receptor type 2 (CCR2); Interferon beta (IFN-β);* Interferon gamma *(*IFN-γ*); tumor necrosis factor alpha (TNF-α); interleukin 1b (IL-1b); interleukin 2 (IL-2); interleukin 4 (IL-4); interleukin 8 (IL-8); interleukin 10 (IL-10); interleukin 18 (IL-18); krüppel-like factor 4 (KLF4); hypoxia-inducible factor 1-alpha (HIF1α)*
