# Supplementary material for: Transfection of Vein Grafts with Early Growth Response Factor-1 Oligodeoxynucleotide Decoy: Effects on Stem-Cell Genes and Toll-like Receptor-Mediated Inflammation
Source: Int J Mol Sci. 2023 Nov 1;24(21):15866. doi: 10.3390/ijms242115866 (PMC10647335; doi:10.3390/ijms242115866)
Supplement: Supplementary file 1 [file ijms-24-15866-s001.zip › Supplemental Table S2. Rabbit target gene.pdf]

Supplemental Table S2. Primers

**Rabbit target gene**	**Forward primer**	**Reverse primer**	**Target size**	**NM**
**b actin**	CCATGTACGTGGCCATCCAG	TCTTCATGAGGTAGTCGGTCAGGTC	148nts	NM_001101683
**TLR2**	CTCCTGCTGACGCTGCTC	TTCCTCGGCTTCCTCTTGG	120nts	Trace Archive
**TLR3**	ATCTCCTCTCTTTGGGGACTGTTG	TGTTGGTGGGCAGGTCATCAGG	125nts	Trace Archive
**TLR4**	CTCACATCCGAGTTGCCTTCCG	AAATGCTCCCTGGTACACCTGTTC-3	125nts	Trace Archive
**TLR8**	ATCTTGTTCTTCTTCTCGTTCTC	CCTGTAACCTCTGACCTTGG	126nts	Trace Archive
**MYD88**	CCCTTTGTCTCTCGACTCTTGG	TACGAGAACAGCCACTGCCC	125nts	Trace Archive
**NFκB**	ATGCCAATGCCCTCTTCGACT	CGTGACTTCCAGCAGATCCCT	122nts	Trace Archive
**CCL4**	GAGACCACCAGCCTCTGCTC	TCAGTTCAGTTCCAAGTCATCCAC	123nts	NM_001082196
**CCL20**	TATCGTGGGCTTCACACAGC	CCATTCCTTCTTCGGATCTGC	115nts	Trace Archive
**CCR2**	GGTTGCTGAGAAGCCTGACACGC	CAGGTCTGTATTCTTCAACAAGCCCTCG	125nts	Trace Archive
**IFNβ**	TCCAACTATGGCACGGAAGTCT	TTCTGGAGCTGTTGTGGTTCCT	133nts	XM_002707968
**IFNγ**	TGCCAGGACACACTAACCAGAG	TGTCACTCTCCTCTTTCCAATTCC	127nts	NM_001081991
**TNFa**	CTGCACTTCAGGGTGATCG	CTACGTGGGCTAGAGGCTTG	133nts	NM_001082263
**IL1b**	TTGAAGAAGAACCCGTCCTCTG	CTCATACGTGCCAGACAACACC	128nts	NM_001082201
**IL2**	GCCCAAGAAGGTCACAGAATTG	TGCTGATTGATTCTCTGGTATTTCC	128nts	NM_001163180
**IL4**	CGACATCATCCTACCCGAAGTC	CCTCTCTCTCGGTTGTGTTCTTG	122nts	NM_001163177
**IL8**	CCACACCTTTCCATCCCAAAT	CTTCTGCACCCACTTTTCCTTG	122nts	NM_001082293
**IL10**	CTTTGGCAGGGTGAAGACTTTC	ACTGGATCATCTCCGACAAGG	126nts	NM_001082045
**IL18**	ACCAAGGACAGCAACCTGTGTT	ACAGAGAGGCTTACAGCCATGC	120nts	NM_001122940
**KLF4**	ACGTACTCGCCTTGCTGATG	CAAAAACCCCGAATTGCCCG	151	XM_017347259.1
**HOXA5**	CATGCTCTTTGCCTCTCCGA	TTAGGGCAACGAGAACAGGG	248	Trace Archive
**NANOG**	TACCCAGGCTTCTACCCTGC	GGTTACTCCACGACTGGCTG	136	XM_002712762.1
**H1F1α**	TTTTGGCAGCAACGACACAG	GTGCAGGGTCAGCACTACTT	173	Trace Archive

*Myeloid differentiation primary response 88 (*MYD88*); TLR: toll-like receptor; Nuclear factor kappa-light-chain-enhancer of activated B cells (NF-κB); C-C chemokine ligand (CCL4); Chemokine (C-C motif) ligand 20 (CCL20); C-C chemokine receptor type 2 (CCR2); Interferon beta (IFN-β);* Interferon gamma *(*IFN-γ*); tumor necrosis factor alpha (TNF-α); interleukin 1b (IL-1b); interleukin 2 (IL-2); interleukin 4 (IL-4); interleukin 8 (IL-8); interleukin 10 (IL-10); interleukin 18 (IL-18); krüppel-like factor 4 (KLF4); hypoxia-inducible factor 1-alpha (HIF1α)*
