# Supplementary figures and images for: Transfection of Vein Grafts with Early Growth Response Factor-1 Oligodeoxynucleotide Decoy: Effects on Stem-Cell Genes and Toll-like Receptor-Mediated Inflammation
Source: Int J Mol Sci. 2023 Nov 1;24(21):15866. doi: 10.3390/ijms242115866 (PMC10647335; doi:10.3390/ijms242115866)

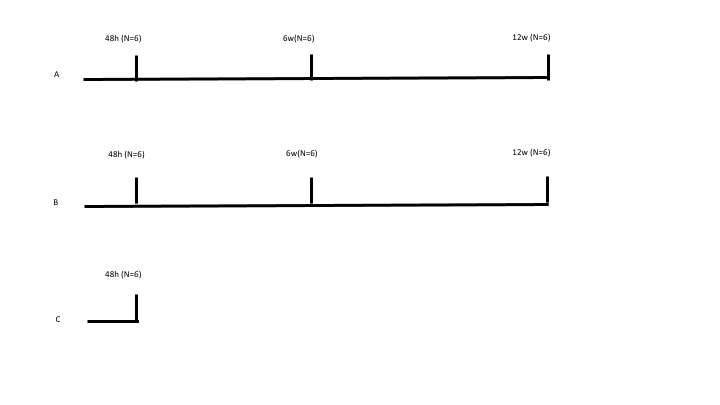

Supplement: Supplementary file 1 [file ijms-24-15866-s001.zip › Supplemental Figure S1. Experimental timeline.tif]
